# Exploring the Effect of the COVID-19 Pandemic on the Dental Team: Preparedness, Psychological Impacts and Emotional Reactions

**DOI:** 10.3389/froh.2021.669752

**Published:** 2021-04-29

**Authors:** Gerry Humphris, Jennifer Knights, Laura Beaton, Marianna Araujo, Siyang Yuan, Jan Clarkson, Linda Young, Ruth Freeman

**Affiliations:** ^1^School of Medicine, University of St Andrews, St Andrews, United Kingdom; ^2^NHS Education for Scotland, Edinburgh, United Kingdom; ^3^Dental Health Services Research Unit, School of Dentistry, University of Dundee, Dundee, United Kingdom

**Keywords:** COVID-19, dental team, burnout—professional, psychology, depressive symptoms, preparedness, impact of events, SEM modeling

## Abstract

**Background:** The COVID-19 pandemic has placed increased demands on clinical staff in primary dental care due to a variety of uncertainties. Current reports on staff responses have tended to be brief enquiries without some theoretical explanation supported by developed measurement systems.

**Aim:** To investigate features of health and well-being as an outcome of the uncertainties surrounding COVID-19 for dentists and dental health professionals in primary dental care and for those in training. In addition, the study examined the well-being indices with reference to normative values. Finally a theoretical model was explored to explain depressive symptoms and investigate its generalisability across dentists and dental health professionals in primary dental care and those in postgraduate training.

**Methods:** A cross-sectional survey of dental trainees and primary dental care staff in Scotland was conducted in June to October 2020. Assessment was through “Portal,” an online tool used for course bookings/management administered by NHS Education for Scotland. A non-probability convenience sample was employed to recruit participants. The questionnaire consisted of four multi-item scales including: preparedness (14 items of the DPPPS), burnout (the 9 item emotional exhaustion subscale and 5 items of the depersonalisation subscale of the MBI), the 22 item Impact of Event Scale-Revised, and depressive symptomatology using the Patient Health Questionnaire-2. Analysis was performed to compare the levels of these assessments between trainees and primary dental care staff and a theoretically based path model to explain depressive symptomology, utilising structural equation modelling.

**Results:** Approximately, 27% of all 329 respondents reported significant depressive symptomology and 55% of primary care staff rated themselves as emotionally exhausted. Primary care staff (*n* = 218) felt less prepared for managing their health, coping with uncertainty and financial insecurity compared with their trainee (*n* = 111) counterparts (all *p*'s < 0.05). Depressive symptomology was rated higher than reported community samples (*p* < 0.05) The overall fit of the raw data applied to the theoretical model confirmed that preparedness (negative association) and trauma associated with COVID-19 (positive association) were significant factors predicting lowered mood (chi-square = 46.7, *df* = 21, *p* = 0.001; CFI = 0.98, RMSEA = 0.06, SRMR = 0.03). Burnout was indirectly implicated and a major path from trauma to burnout was found to be significant in primary care staff but absent in trainees (*p* < 0.002).

**Conclusion:** These initial findings demonstrate the possible benefit of resourcing staff support and interventions to assist dental staff to prepare during periods of high uncertainty resulting from the recent COVID-19 pandemic.

## Introduction

Reports of anxieties concerning litigation, fears for family welfare and general uneasiness surrounding the impact of the COVID-19 pandemic on dental practice have recently been noted in dentists from cross-sectional surveys conducted in various countries [1, 2]. A recent survey in practicing dentists in Pakistan has shown that 75% were fearful about “getting the infection” [3]. Staff are caught in a clinical dilemma. That is, maintaining the same high quality care in parallel with the increased threats of infection and reducing standards. Dental teams have relied upon clinical guidelines and Personal Protective Equipment (PPE) to reduce, partially, their anxieties of transmitting COVID-19 [4, 5].

Work in the wider health environment suggests that many health practitioners experience anxiety and depression associated with patient management, conflict and communication difficulties resulting in acute stress disorder during this time [6]. Other reports point to a similar pattern of emotionality in the face of providing quality care during the current COVID-19 pandemic [7, 8]. Focusing on the “ongoing uncertainty” and reliance on PPE, Albott et al. [9] proposed that the elements of providing health care during COVID-19 was analogous to “battlefield conditions.” Moreover, the outcome of repeated stressful encounters, was stress inoculating. In their opinion, being physically and emotionally prepared for practice during periods of high uncertainty such as the current pandemic could, paradoxically, reduce anxiety and improve coping. Hence there is lack of clarity in the response of health care personnel such as dental professionals over the course of this COVID-19 pandemic.

Based on the above research, important factors including repetitive experience of uncertainty, together with preparedness, could promote or inhibit adverse and long-lasting emotional effects [10, 11]. Adopting the “battlefield” analogy further [9], it may be proposed that the emotional effects of providing patient care during COVID-19 could trigger elements of psychological trauma. These observations and suppositions are supported by work in America and Australia where health professionals experienced anxiety and occupational pressures as a consequence of COVID-19 [12, 13]. What remained unclear, however, was how the impact of COVID-19, together with uncertainties, affected the health and well-being of dentists and dental health professionals in primary dental care and postgraduate training.

There is an urgent need to investigate the impact of COVID-19 and the resulting uncertainty since this would greatly assist our understanding of who reacts to the situations they are located in, who appears vulnerable and how additional support and training might be designed and delivered.

**Figure 1:**
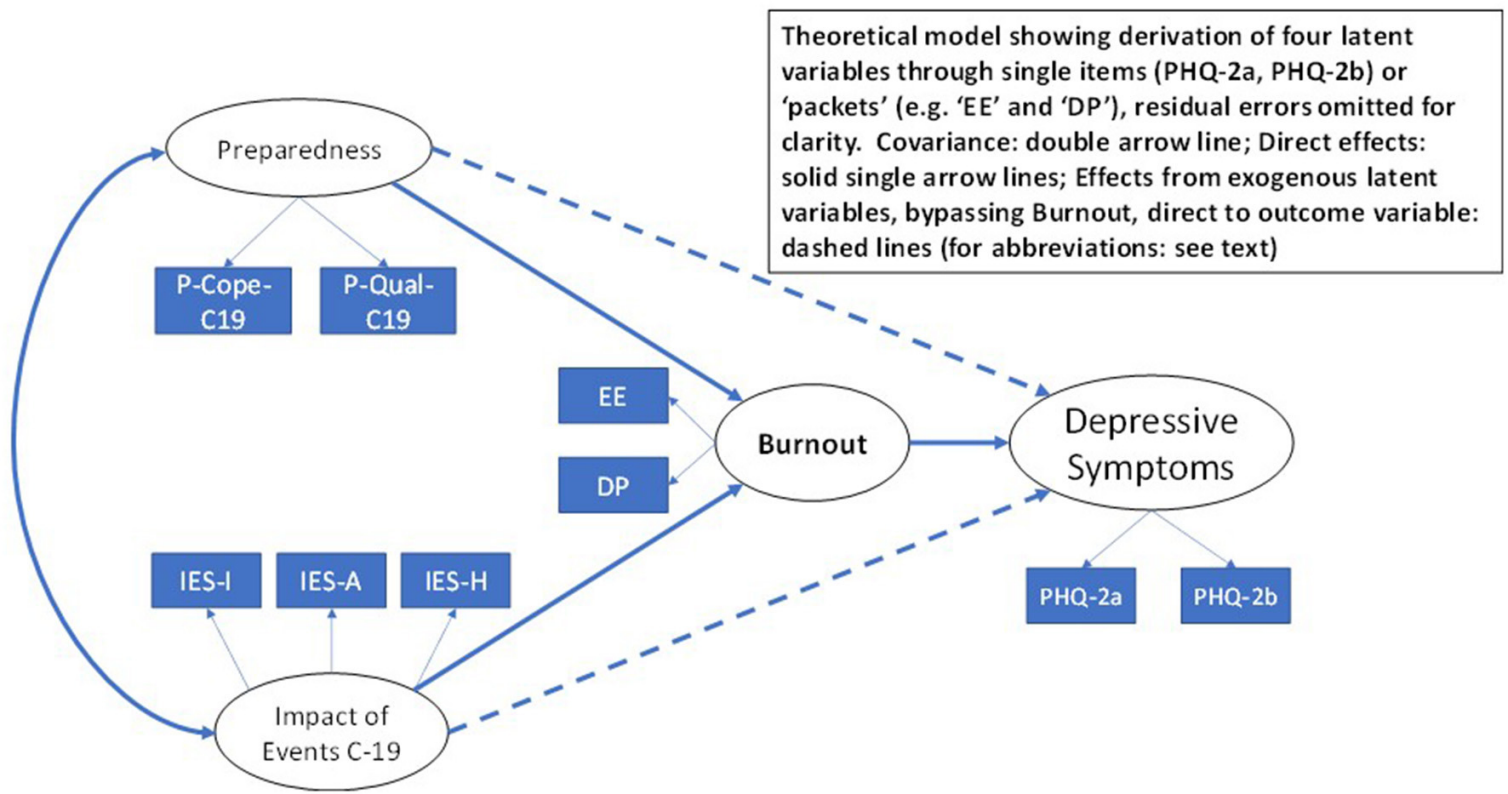
Conceptual model.

The aim of this research was to investigate features of health and well-being as an outcome of the uncertainties surrounding the impact of COVID-19 for dentists and dental health professionals in primary dental care, and for those in training, using a cross-sectional design. Our working hypothesis was based upon a theoretical model that proposes that the impact of the COVID-19 pandemic on the working and training environment influences a pattern of burnout that in turn results in an increased experience of depressive symptoms (Figure 1). The model presented has been derived from a previous European 5 year longitudinal study of dental students [14] and features the detailed examination of burnout predicting depression [15]. The strongest evidence of burnout being responsible for depressive symptoms over time has been reported by the three-wave 4 year longitudinal study of a national sample of Finnish dentists [16].

Specifically, we surveyed dentists and dental health professionals in primary dental care and those in postgraduate training in Scotland to report levels of preparedness for COVID-19 related care issues, trauma associated with the COVID-19 pandemic, burnout and depressive symptomology. Further we compared levels of the psychological constructs: trauma associated with the COVID-19 pandemic, burnout and depressive symptomology across dentists and dental health professionals in primary dental care and those in postgraduate training and refer to normative values. Finally we explored a theoretical model to explain depressive symptoms and investigate its generalizability across dentists and dental health professionals in primary dental care and those in postgraduate training.

## Methodology

### Study Design

A cross-sectional design was employed.

### Setting

This research was carried out in Scotland where National Health Service Dentistry is a devolved matter and responsibility for oral health and dental provision therefore rests with the Scottish Government. Postgraduate dental training is organised and administered by NHS Education for Scotland (NES) on a Scotland-wide basis. NES is the main provider of training programmes for dental nurses and delivers continuous professional development for dentists, dental care professionals and practice teams in order that they keep up to date with best practice and are able to maintain their registration with the General Dental Council.

### Sample Size

To determine an appropriate sample size for the three objectives we argued that group differences between staff groupings would be confirmed with a small to medium effect size (0.35) with a sample of approximately 260 participants on our outcome variable namely: depressive symptomology Alternatively, an additional power analysis indicated that with a sample size of 300 participants and a multi-variable linear model that explains 40% of the variance of the outcome (predicted say with seven covariates) the ability to detect an improvement of R square of 2.5% with the inclusion of a further covariate would be detected reliably employing a conventional alpha level of 5% with 80% power. For structural equation modelling, the convention is to run group comparisons with no lower samples than 100 per group. Our minimum sample size to run this type of analysis would be 200 participants.

### Sample

A non-probability convenience sample [17] was employed to recruit participants. The sample included vocational dental practitioners, vocational dental therapist hygienists and trainee dental nurses and all members of the dental team in primary care outlined in detail below. Undergraduate and postgraduate dental students, and all dental staff in the hospital sector were excluded. Specifically, the survey was open for trainees to respond over a period of 6 weeks from mid-June to end of July 2020. By August 2020 dentists and dental health professionals in primary dental care settings in Scotland, who had a NHS Education for Scotland (NES) Portal account (and had opted to receive marketing communications) were notified of the opportunity to participate. The survey was open for dentists and dental health professionals in primary dental care settings to respond from mid-August to early October 2020. Two reminders were sent at 2 week intervals.

### Questionnaire

The questionnaire consisted of six sections. The last section inquired of the respondent's dental professional group, service area, type of practice (where applicable), re-deployment status and demographics (age, sex) including a free-response comment box.

The remaining sections continued the following psychometric measures:

1. Emotional Exhaustion, consisting of 9 questions and
2. Depersonalisation consisting of 5 questions. These two measures are two subscales from the Maslach Burnout Inventory [14, 18];
3. Patient Health Questionnaire−2 (PHQ-2) is a two item screening questionnaire utilised to assess depressive symptomology [19]. The stem of the scale was: “Over the last 2 weeks, how often have you been bothered by any of the following problems:” The items were worded: “Little interest or pleasure in doing things” and “Feeling down, depressed or hopeless” on an answering scale: “Not at all” (1), “Several days” (2), “More than half the days” (3) and “Nearly every day” (4)
4. Dental Professional Preparedness for Practice Scale (DPPPS). This scale consisted of 14 items that were derived from the original designed for medical service personnel [12]. A full description of the items and factorial structure is provided in a Supplementary Table 1 (DPPPS Psychometrics). Two sub-scales (named: P-Cope-C19 and P-Qual-C19) were created from the items which had the stem: “In the current COVID-19 pandemic, how well are you prepared for:” Respondents checked one rating from the five available categories: “Unprepared” (1), “not well-prepared” (2), “prepared” (3), “well-prepared” (4), “extremely well-prepared” (5). The sub-scales were divided by the number of items comprising the sub-scale to derive a score ranging from 1 to 5. This enables comparison with the original published measure [12].
5. Impact of Event Scale-Revised comprises of 22 items with three subscales to assess Intrusions (8 items), Avoidance (8 items) and Hyperarousal (6 items). It is a well-recognised assessment that describes the three major features of post-traumatic stress disorder (PTSD) [20]. The wording of the scale was identical but with the addition of participants to complete: “with reference to the COVID-19 pandemic and effects to your training or on your workplace.”
6. All comments that were included as text in the open-ended dialogue box on-line were tabulated and read by the authorship.

### Administration of the Questionnaire

The survey was distributed electronically to all participants. Invitations were sent to email addresses associated with the NHS Education for Scotland (NES) Portal accounts of those in the primary care setting who had selected the professional groups upon their registration with Portal. Portal is an online tool used for course bookings/management administered by NES. The trainees were invited to take part through their relevant training hub, with support from the NES Dental Dean.

### Ethical Considerations

Ethical approval was provided (18^th^ May 2020) by the University of Dundee Nursing and Health Sciences and Dentistry Research and Ethics Committee (Reference: UOD\SDEN\STAFF\2020\013-Freeman). A Participant Information sheet containing detailed information about the study was provided, and written consent was collected from all participants.

### Statistical Analysis

Detailed inspection of the measurement properties of the self-report measures was made to determine reliability and unidimensionality of the scales. Cronbach alpha coefficients were calculated for each psychological construct and visual inspection made of these values with published estimates in key original measurement reports. Descriptive analyses (means, SDs and medians) were run and breakdown of scale scores by the main professional grouping was performed. Inference testing via the *t* statistic was inspected between the 2 professional groups. In addition, the total scores of those measures were compared to published normative values, with the exception of preparedness for practice (DPPPS). This measure was derived from items designed for medical trainees and therefore norms were not available.

Three latent variables, namely: trauma, preparedness and burnout, were derived from item packets [21], and the two raw items to construct the outcome latent variable: depressive symptomatology [22]. The structural equation model (*sem* procedure in Stata) was tested including all possible paths and allowed to converge using the maximum likelihood estimator [23]. By convention to enable equation identification one item packet or raw variable for each latent variable was constrained to unitary variance thereby “setting” the scale. The remaining other item packets or raw variables were allowed to estimate freely. Correlations between residual errors on all items were inspected to check for independence. Standard overall goodness of fit statistics (Chi square, CFI, RMSEA, SRMR) were utilised to assess correspondence of raw data to the hypothesised model [24, 25]. The analysis was planned to run simultaneously for both the trainee and primary care staff groups. Overall test of group invariance (*ginvariant* option) was conducted, *a priori*, to determine if the paths between the latent variables were comparable across the two groups, revealed through inspection of z scores.

## Results

The sample included Vocational Dental Practitioners (151, 36% of total approached), Vocational Dental Therapists (11, 3%), Dental Core Trainees (89, 21%) and Dental Specialty Training Registrars (38, 9%) undertaking their training in Scotland during the month of June 2020. This was extended to include Trainee Dental Nurses (118, 29%) and Trainee Orthodontic Therapists (7, 2%). The number of questionnaires that were appropriate for analysis was 329. A number of participants (*n* = 5) failed to supply consent leaving the proportion of useable data at 98.5%. The response rate for the trainees was 27% (111/414) of all trainees in Scotland on a NES training programme. Not all primary dental staff possess a NES Portal membership hence a response rate was not calculated.

The breakdown of staff showed a much younger age profile in the trainees (mean 26 vs. 43 years of age, *t* = 14.93, *df* = 298, *p* = 0.0001) as expected (Table 1). The degree of deployment in the primary care compared to the trainee group was significantly different (chi-square = 8.80, *df* = 1, *p* = 0.003) at 20 vs. 7 percent respectively. Seventy-six percent of the primary care staff stated that they were mostly or fully employed within NHS practice. Eighty one percent of the sample were female. A full breakdown of the specialties of the sample are available in the Supplementary Table 2 Professional Group Membership. The internal consistency reliability estimates (Cronbach alphas) are presented in Supplementary Table 3.

**Table 1:** Demographics, practice composition and deployment status.

		**Trainee**		**Primary care**		**Total sample**	
		**n**	**%**	**n**	**%**	**n**	**%**
Sex	Male	23	21	40	18	63	19
	Female	87	79	177	81	264	81
	Prefer not to say	0	0	1	0	1	0
Age	Mean (SD) years	26 (5)		43 (11)		37 (12)	
Practice Type	Fully NHS			67	31	67	31
	Mostly NHS			97	45	97	45
	Equal NHS/Private			32	15	32	15
	Mostly Private			19	9	19	9
	Fully Private			1	0	1	0
Re-deployed	Yes	8	7	43	20	51	16
	No	103	93	175	80	278	84

Levels of preparedness were examined in two ways. First, the aggregate sub-scale values for the two factors labelled coping (P-Cope-C19) and quality (P-Qual-C19) were reviewed (Table 2). Trainees showed greater preparedness compared with the primary care staff (*p* < 0.001). Second, on closer inspection of the five questions that constitute the Cope sub-scale by frequency in each staff group (Table 3), it is evident that the primary care professionals are less prepared than their trainee counterparts in looking after their health, that is 37 vs. 15% respectively, and coping with uncertainty, that is 48 vs. 38% (both sets of proportions, *p* < 0.05).

**Table 2:** Means (SDs) medians, and percent above cut-off of psychological constructs across the two major staff groupings with *t* statistics and *p* levels.

	**Dental trainee**			**Primary dental care**			**Total sample**			**Trainee vs. primary care**	
	**Mean**	**SD**	**Median**	**Mean**	**SD**	**Median**	**Mean**	**SD**	**Median**	***t***	***p***
Preparedness^†^											
Prepared for C-19	2.29	0.60	2.2	2.04	0.66	2	2.12	0.65	2.1	3.37	**<0.001^#^**
Prepared to give quality care	3.73	0.72	3.71	3.58	0.72	3.57	3.64	0.69	3.57	1.82	0.07
Impact of Events due to C-19											
Intrusiveness	0.98	0.82	0.75	1.15	0.91	1.00	1.09	0.88	0.88	−1.62	0.11
Avoidance	1.04	0.83	0.88	1.09	0.84	0.94	1.08	0.84	0.88	−0.54	0.59
Hyperarousal	0.81	0.85	0.50	1.05	0.95	0.83	0.97	0.92	0.67	−2.18	**0.03**
Total Score	0.95	0.78	0.77	1.08	0.84	0.91	1.04	0.82	0.86	−1.34	0.18
(percent above cut-off)	27.5%			32.3%			30.4%				
Burnout Scales											
Emotional Exhaustion	22.32	10.11	21.00	31.70	16.31	28.00	28.53	15.17	25.00	−5.49	** <0.001**
(percent above cut-off)	25.7%			55.6%			45.5%				
Depersonalisation	7.74	3.45	7.00	9.00	5.24	7.00	8.57	4.74	7.00	−2.29	**0.02**
(percent above cut-off)	18.2%			32.2%			27.5%				
Depressive Symptoms	1.83	1.50	2.00	2.03	1.76	2.00	1.96	1.68	2.00	−1.02	0.31
(percent above cut-off)	25.2%			28.8%			27.6%				

† **No cut-off values on this this scale*.*

# *significant differences in bold*.

**Table 3:** Frequency breakdown of preparedness of P-Cope-C19 items across trainee and primary care staff groups.

	**Trainees**			**Primary care**				
**During COVID_19 pandemic…**	**N**	**Percent**	**Cumulative %**	**N**	**Percent**	**Cumulative %**	**chi-sq (*df*4)**	***p***
**Managing your health including stress**							20.04	0.001
unprepared	3	2.7	2.7	16	7.3	7.3		
not well prepared	14	12.6	15.3	63	28.9	36.2		
prepared	50	45.1	60.4	90	41.3	77.5		
well prepared	38	34.2	94.6	41	18.8	96.3		
extremely well prepared	6	5.4	100.0	8	3.7	100.0		
**Coping with uncertainty in general**							9.87	0.04
unprepared	5	4.5	4.5	32	14.3	14.3		
not well prepared	37	33.3	37.8	73	33.6	47.9		
prepared	42	37.8	75.7	76	35.0	82.9		
well prepared	24	21.6	97.3	31	14.3	97.2		
extremely well prepared	3	2.7	100.0	6	2.8	100.0		
**Coping with uncertainty about future and career job prospects**							6.04	0.19
unprepared	14	12.6	12.6	45	20.6	20.6		
not well prepared	44	39.6	52.3	94	43.1	63.8		
prepared	31	27.9	80.2	51	23.4	87.2		
well prepared	19	17.1	97.3	23	10.6	97.7		
extremely well prepared	3	2.7	100.0	5	2.3	100.0		
**Coping with financial insecurities**							11.75	0.019
unprepared	9	8.1	8.1	42	19.4	19.4		
not well prepared	43	38.7	46.9	71	32.9	52.3		
prepared	31	27.9	74.8	68	31.5	83.8		
well prepared	23	20.7	95.5	25	11.6	95.4		
extremely well prepared	5	4.5	100.0	10	4.6	100.0		
**Understanding the purpose and practice of appraisal**							18.24	0.001
unprepared	1	0.9	0.9	17	7.9	7.9		
not well prepared	20	18.0	18.9	62	28.2	36.1		
prepared	51	46.0	64.9	88	40.7	76.9		
well prepared	35	31.5	96.4	39	18.1	94.9		
extremely well prepared	4	3.6	100.0	11	5.1	100.0		

The total scores for the IES total scale (M = 1.04, SD = 0.82), Burnout sub scales: emotional exhaustion (EE) (M = 28.5, SD = 15.2), and depersonalisation (DP) (M = 8.57, SD = 4.74), and depressive symptomatology (M = 1.96, SD = 1.68), were examined according to recognised published cut-off values (Table 2). The EE proportion of respondents who scored at cut-off and above was 45%, and the Impact of Event Scale total indicated that 30% of the whole sample of respondents were above the recognised normative standard. The levels of depersonalisation and depressive symptomatology was 27% for both measures. Attention was drawn particularly to the 54% level of emotional exhaustion at cut-off and above, for the primary care staff. Mean aggregate comparisons across the two major staff groupings confirmed that the primary care staff were significantly more emotionally exhausted, adopting greater depersonalisation and reporting higher hyperarousal compared to trainees.

The structural equation model as represented in Figure 1 was run including the group categorisation of trainee and primary care staff included. The model was estimated (maximum likelihood) with parameters obtained simultaneously across both groups. The correlation matrices of raw data for the total sample and separately for the two groups are presented in Supplementary Table 4. The analysis was divided into a discrete number of steps as recommended [26].

**Figure 2:**
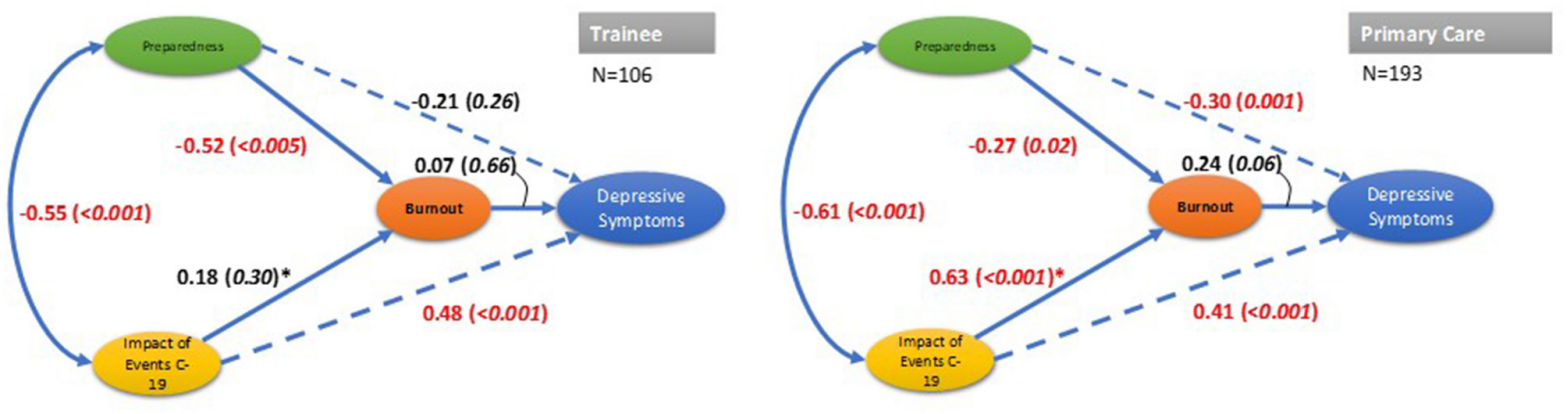
Result of path models for trainee and primary care staff groups (standardized coefficients presented with *p* values in brackets). Overall model fit statistics, Chi-square(d*f* 54)=95.82, *p* = 0004; CFI=0.97; RMSEA=0.072, 95%CIs: 0.48, 0.95; Group fit statistics: SRMR=0.006 (Trainee), 0.038 (primary care); ^*^Association significantly different between groups: chi-square (d*f* 1)=11.95, *p* = 0.005. Indicator raw variables omitted to improve legibility; significant associations in red font.

First, the check that the model would run was shown by convergence in 16 iterations and there were no unexpected coefficient values returned. We confirmed that the estimation procedure returned nothing to alert a rogue analytical procedure and no Heywood cases were reported [27]. Of interest were the associations between the latent variables. This analysis enabled the inspection of the model coefficients and respective standard errors for each group. For ease of comparison these are presented as correlations (standardized estimates), and z scores were calculated using robust standard errors to provide indicators of statistical significance. The overall fit was very good as expressed by the fit indices including chi-square = 95.8, *df* = 54, *p* = 0.0004, and CFI = 0.974, RMSEA = 0.07, 95%CIs 048, 0.95, SRMR = 0.054 (Figure 2).

Second, group invariant Wald chi-square tests showed that a single path was clearly different between the two groups (Wald chi-sq = 11.9, *df* = 1, *p* < 0.0005), namely the IES and burnout latent variables path.

Lastly, we ran, for completeness, a further model with no group categorisation and the fit with this more parsimonious approach was excellent, requiring 3 iterations (chi-square = 46.7, *df* = 21, *p* = 0.001; CFI = 0.984, and RMSEA = 0.06, 95%CIs 0.039, 0.089), SRMR = 0.031.

A large amount of text was also included in the survey responses. These responses (119 in total) were transcribed into a single document and three verbatim quotes were selected by members of the research team (JK, GH, and RF) to assist illustration of our main findings in greater detail.

The first quote, from an experienced hygienist lucidly expresses anxiety and sadness from low professional recognition with feelings of hopelessness, and lack of control. Avoidance is recognised as a strategy this is not realistic to be acted upon.

- Quote 1: “Never have I felt so undervalued in the 30 years qualified. I have given so much to prevention in dentistry and the uncertainty of my future career has caused much anxiety and sadness. I have been concerned about patients' oral health but have not been allowed to contact and reassure them. I feel totally helpless in this situation although I feel I could do more. To not be in control of ones' decisions throughout this has been extremely difficult. Part of me wants to walk away but as an experienced professional… I cannot.” (Hygienist)

The second quote gives a similar impression of being misunderstood by the media and the public and generally poorly valued. The current pandemic has resulted in great pressure on the individual and illuminated strains in the systems operating in the delivery of dental care. Little mention is made of having information or strategies that might have enabled this individual to prepare for such a scenario.

- Quote 2: “This has been one of the most difficult times in my life so far. The fact that we have been forced to stop working has put huge strain on both my personal and working life. I never ever thought that as a dentist that my career would be in jeopardy. It has made me question my entire career choice and made me very much regret putting so much energy and study into the career I've chosen. Our entire field has been shown how undervalued and excluded we are from other professions. We are the last to be considered and we get little sympathy from the general public as dentists are demonised in the media. We are seen as sadists with little financial worries and this is far from the truth. I have spent this entire time worrying about my patients and how their lack of treatment will impact on their future dental health and how we are likely the foot the blame and the bill on what their needs will be. I also feel sad that a two-tier system has been put in place regarding providing private dental treatment to patients and again, this has put dentists in a tricky position which again makes them look bad to the public. Greedy dentists who put making money over providing treatment to their patients at a loss. The whole pandemic has really shown the true colours of the system that we work in and as these restrictions are prolonged, the worse the situation is getting, both financially for practices and dentists, and ethically as patients “treatment need is being neglected.” (Dentist GDS)

The third quote repeats some of the main issues previously expressed but raises disturbingly the consequences for this individual in trying to manage in a threatening situation. The effects of running their business with the realistic chance of financial ruin appears to be averted through central government assistance, and the scenario briefly outlined is of striking relevance to this study and the attempt to understand the personal implications that many respondents were facing.

- Quote 3: “This survey has highlighted the despair, hopelessness and uncertainty I feel for my future and ability to cope with such a shocking and uncontrollable change to my financial and working situation. I have suicidal thoughts on a daily basis, but the only reason I am able to function is the business bounce back loan I have (which) my new accountant told me was available.” (Dentist GDS)

## Discussion

This survey was launched to potential participants within Scotland during the COVID-19 pandemic, at a time of high uncertainty of clinical provision when a sizeable proportion of clinicians were re-deployed. The response rate by the trainees was ~27% of all trainees registered in NES training programmes in Scotland. The number of primary care staff that participated was limited, as according to national statistics there were 2,801 dentists and this sample included, in addition nurses, therapists and dental hygienists. Hence, we obtained a reasonable sample size for quantitative analysis that satisfied our power calculations but cannot claim this was a representative sample.

The overall aim of this study was to provide a snap-shot of the well-being of members of the dental trainee staff and primary dental care staff, as well as some detail of the degree of preparedness to manage the rigours of the COVID-19 pandemic and routine clinical matters. Previous rapid reports providing some reflections of staff reactions to the pandemic have been limited in scope and detail, unfortunately, relying on brief assessments and somewhat narrow descriptions. The approach adopted in this study has been to utilise measures that enable comparison to previous work and normative data sets, as well as providing a more comprehensive offering that includes, not only an outcome assessment of depressive symptomology, but also the intervening variable of burnout and predictor variables of preparedness and psychological reaction (i.e., trauma) associated with the COVID-19 pandemic.

The preparedness of staff to the pandemic varied across the groups. Notable was the discrepancy related to managing “health and stress” as well as “uncertainty” across trainee and primary care staff. Substantially above a third of primary care staff (36%) were not prepared in taking care of their health and stress levels compared with trainee staff (15%). Unsurprisingly, over half of primary care staff acknowledged that they were not prepared financially for the effects of the pandemic. Although there were no normative cut-offs for the preparedness measure, it is interesting that the mean levels reported for junior doctors following their undergraduate training ranged from 3.6 to 4.4 [12]. The preparedness mean values of the staff included in this study ranged from 2.1 for the coping with COVID-19 and 3.6 for care quality during the pandemic. It can be proposed that there is substantial improvement in preparedness that can be developed through increased training.

Not only are primary care staff less prepared during the pandemic relative to the trainees but they also express greater emotional exhaustion. The primary care staff percentage scoring at, or above the cut-off (55%) was over double the rate of trainees (25%). A smaller discrepancy, although still significant, was reported for depersonalisation. The survey did not contain the third sub-scale: personal accomplishment, in order to ration the item pool of the whole survey questionnaire. In a mapping exercise to understand the meaning associated with the raw scores of the EE and DP scales in US physicians using Item Response Theory (IRT) methodology [28] it may be inferred from the IRT estimates that our respondents who scored at the mean level (i.e., 28), of the total sample including all staff in the survey, would endorse feeling “*fatigued”* or “*burned out”* at least once a week and furthermore for those at a level of emotional exhaustion at 1 standard deviation above the mean (i.e., 43) endorse a weekly frequency of being “*at the end of my rope”*. These levels deserve close attention, hence the association with other psychological responses in the model presented are now considered.

Depressive symptomology was 27% across the sample which compares with 18% in a population-based cohort in normal conditions [29]. A more recent comparison during the first phase of the COVID-19 pandemic “lockdown” with a representative UK sample (*N* = 2,025) survey using the related PHQ-9 found the UK sample to exhibit a significantly lower level of depressive symptomology (chi-square = 4.97, *df* = 1, *p* < 0.03), that is, 22% were rated at cut-off or above [30]. An aggregate statistical test showed a significant higher mean level of the PHQ-2 depression index in the current dental respondents compared with the Caneo et al. community sample (*t* = 7.17, *df* = 5094, *p* < 0.0001). The PHQ-2 does not confirm that the respondent scoring at or above cut-off, was necessarily clinically depressed, however the individual with a cut-off score, or above, shows features of the depressive condition. Within a community there will be an underlying rate of these symptoms regardless of current health threats such as the COVID-19 pandemic. However, the small number of responses filtered from the free responses illustrates that symptoms were freely expressed and non-trivial. The broad finding of the sample collected in this study showed a raised level of depression symptomology to what would be expected.

The third verbatim free response (Quote 3) presented in the results contains a revealing disclosure of suicidal ideation as frequently as daily during the pandemic. In comparison, it is interesting to find from a national UK study the report over 3 monthly waves from March to May 2020 of respondents' suicidal ideation of ~8–10 percent of at least one day in the preceding week of the enquiry [31]. The important detail to be revealed in the O'Connor survey was the significant increase in reporting of suicidal ideation over the 3-month period. Our study did not include a specific question on suicidal ideation and therefore cannot provide a more quantitative comparison, however the non-solicited volunteering of this disclosure in the free-response comments of our survey instrument gives cause for concern.

Overall model fit from both staff groups was judged to be good from inspection of the various indices (Chi-square, CFI etc.) and residual co-variance matrices. All paths showed close approximation between the latent variables across staff grouping, with one exception. Of interest, in particular, was the significant variation in the path of Impact of COVID-19 trauma and burnout between trainee and primary care staff. The primary care group showed a very strong significant association reflecting the traumatic impact, possibly the experience of hyper-arousal as indicated by the raised level in these respondents relative to their trainee counterparts.

Unexpectedly, the link between burnout and depression was not confirmed when degree of preparedness and the traumatic impact of COVID-19 was included. A previous extensive longitudinal study, albeit in Finnish dentists, showed the best predictor over two sets of follow ups, each of 2-year duration, of depressive symptomology was burnout [16]. The raw correlations in our study show that burnout subscales and PHQ-2 items are reliably related (*r*'s > 0.3) however the controlling for preparedness and trauma impact (as “third” variables) attenuated this burnout relationship with depression. Our result in the present cross-sectional study is not inconsistent with other more recent reports that have confirmed that the link between burnout and depression, although detectable, is not straightforward [32, 33]. Hence our model requires extension with longitudinal data to fully appreciate the dynamics of how burnout relates to depression.

We are aware of the recent work in the UK [34] who have studied stress and burnout in UK dentists. They adopted path modelling to illuminate the link between these two psychological constructs. Although the analysis they present broadly supports our findings we view with interest and some caution that their supposition that stress “*causes”* burnout. A cross-sectional study cannot easily demonstrate causal processes. Therefore, we believe that researchers need to approach these sophisticated statistical models with caution. Our synthesis portrayed in our theoretical model and data presentation is illustrative at best and requires additional data collection preferably with longitudinal and experimental designs.

### Strengths and Limitations

This study presented a theoretical model consisting of a coherent network of associated psychological constructs as opposed to investigating the simple link between the reaction of the COVID-19 pandemic and a single indicator of mental health. A latent variable methodology to account for measurement error was employed that also enabled detailed checks of compatibility between raw data and the operationalized theoretical model. The analysis strategy employed an overall comparison of the model between trainee and primary care staff, thereby removing multiple testing and risking Type II errors. The resultant model did not require lifting of constraints of independence of residual errors in the measures employed, increasing confidence in overall model parsimony. Finally, the survey included an investigation of the staff preparedness to the pandemic. This has enabled, in the authors' opinion, an important focus for intervening to assist staff in addition to assessing the negative psychological effects of COVID-19.

The study possesses some limitations. First, the survey was based on a cross-sectional design and unable to state firm conclusions on direction of effects. The model tested was hypothetically based upon results obtained by a larger European undergraduate longitudinal study and theoretical rationale [14]. The authors acknowledge that other models could have been fitted which may produce a more salient solution. Second, the authors are also aware of some of the anecdotal responses in the open-ended comment box that participants had a large variety of experiences according to locality or type of workplace across Scotland. The reporting of mean levels and associations across groups diminishes wide ranges of individual opinions and emotional content. A further report of a parallel focus group study is being prepared that reviews the qualitative component of individual's experience in detail. Third, and related, there is awareness that the pandemic has various phases according to virus infection rates and public health restrictions on movement and service provision. The cross-sectional nature of this survey study was unable to plot respondents' opinion changes. Finally, the results need to be treated with caution especially when attempting to generalise beyond the sample collected. The authors believe that these findings are important as they raise concern over the well-being status of the participants included in this study.

### Future Work

This descriptive profile will enable a detailed analysis of the variation of response and aid identification of the potential avenues of intervention and training. The findings will be a platform to design an integrated programme of support that can be tested in future redeployment scenarios. The authors are mindful that staff reactions to the current pandemic are dynamic due to the ever-changing situation of restrictions, case level, discovery of new virus variants and vaccine development and administration. To increase our understanding of staff well-being, a different methodology is required that frequently taps into the expression of distress and coping to model change. Such systems of assessing multiple waves of individual responses are well developed in other fields, for example in studying family relations over time [35, 36]. The authors have conducted an explorative study using weekly diaries of fatigue in a sub-sample of these staff and a manuscript is under development. Further studies are required of intensive longitudinal follow-ups of staff cohorts to appreciate the variable nature of individual reactions to long-term pressurised clinical environments.

## Data Availability Statement

The datasets presented in this article are not readily available because Ethical review board instructed that the data collected remains within the NHS Education Scotland repository and jurisdiction. Requests to access the datasets should be directed to sdpbrn@nes.scot.nhs.uk.

## Ethics Statement

The studies involving human participants were reviewed and approved (18th May 2020) by the University of Dundee Nursing and Health Sciences and Dentistry Research and Ethics Committee (Reference: UOD\SDEN\STAFF\2020\013-Freeman). The patients/participants provided their written informed consent to participate in this study.

## Author Contributions

RF, GH, and JC conceived the study. JC secured support for the study. All authors contributed to the study design. GH led the statistical analyses. GH, RF, and JK led the drafting of the manuscript. All authors contributed to the various drafts and approved the final manuscript for submission.

## Conflict of Interest

The authors declare that the research was conducted in the absence of any commercial or financial relationships that could be construed as a potential conflict of interest.
