# Supplementary material for: Exploring the Effect of the COVID-19 Pandemic on the Dental Team: Preparedness, Psychological Impacts and Emotional Reactions
Source: Front Oral Health. 2021 Apr 29;2:669752. doi: 10.3389/froh.2021.669752 (PMC8757713; doi:10.3389/froh.2021.669752)
Supplement: Supplementary file 1 [file Table_1.DOCX]

Supplementary File Table S1: DPPPS Psychometrics

This brief file explains the derivation and design of the fourteen items that were included in the self-completed questionnaire for participants to complete. The 14 items are listed below in the table in order of appearance in the questionnaire.

|  |  | Factor 1 | Factor 2 |
| --- | --- | --- | --- |
| Item 1 | Managing your health including stress | 0.193 | 0.764 |
| Item 2 | Coping with uncertainty in general | 0.190 | 0.826 |
| Item 3 | Coping with uncertainty about future and career job prospects | 0.101 | 0.685 |
| Item 4 | Coping with financial insecurities | 0.040 | 0.633 |
| Item 5 | Understanding the purpose and practice of appraisal | 0.365 | 0.590 |
| Item 6 | Engaging in self critique of practice and clinical encounters | 0.453 | 0.486 |
| Item 7 | Engaging in self-directive life-long learning | 0.403 | 0.445 |
| Item 8 | Coping with GDC governance and training requirements | 0.501 | 0.376 |
| Item 9 | Maintaining good quality of care | 0.716 | 0.175 |
| Item 10 | Taking part in clinical governance | 0.643 | 0.256 |
| Item 11 | Reducing the risk of cross-infection | 0.763 | 0.120 |
| Item 12 | Organisational decision making | 0.680 | 0.232 |
| Item 13 | Ensuring patient safety | 0.828 | 0.125 |
| Item 14 | Reporting and dealing with error and safety incidents | 0.698 | 0.185 |

Note: Items 6 & 7 deleted from scale following factor analysis (maximum likelihood estimation) with rotation. These two items load on both factors and describe in their wording the process of engaging indicating a possible systematic (linguistic) bias in approach by participants. Hence items 6 & 7 were removed.

A test to identify the factorial structure was performed using Horn’s Parallel factor analysis to validate the presence of 2 factors over a simple unidimensional solution or alternatively a more detailed set of factors, that is 3 or more identifiable constructs within the items. The results of the analysis were clear, demonstrating the identification of a two factor solution. The graph (below) shows that the eigenvalues plotted (scree test) of the first two factors lie well above the eigenvalues estimated from multiple samples (n=2000) of the item responses which have been randomly prepared. Note that the more conservative ‘adjusted’ set of eigenvalues (coloured black lines) from the item pool are higher than the random values (blue line). This approach is preferred to the usual Kaiser Unity eigenvalue rule for selecting the number of underlying factors. The table of loadings, reliability coefficients and item wording are tabulated further below:

|  | Item wording for DPPPS | Cronbach’s alpha | Prepared for coping with C-19 | Prepared for delivery of quality care |
| --- | --- | --- | --- | --- |
|  | *Short DPPPS sub-scale name****: P-Cope-C19*** |  | | |
| Item 1 | Managing your health including stress | **0.845** | 0.196 | **0.781** |
| Item 2 | Coping with uncertainty in general |  | 0.192 | **0.865** |
| Item 3 | Coping with uncertainty about future and career job prospects |  | 0.112 | **0.683** |
| Item 4 | Coping with financial insecurities |  | 0.055 | **0.621** |
| Item 5 | Understanding the purpose and practice of appraisal |  | 0.346 | **0.550** |
|  | | | | |
|  | *Short DPPPS sub-scale name****: P-Qual-C19*** |  | | |
| Item 8 | Coping with GDC governance and training requirements | **0.879** | **0.466** | 0.343 |
| Item 9 | Maintaining good quality of care |  | **0.697** | 0.174 |
| Item 10 | Taking part in clinical governance |  | **0.610** | 0.239 |
| Item 11 | Reducing the risk of cross-infection |  | **0.782** | 0.126 |
| Item 12 | Organisational decision making |  | **0.673** | 0.234 |
| Item 13 | Ensuring patient safety |  | **0.860** | 0.139 |
| Item 14 | Reporting and dealing with error and safety incidents |  | **0.706** | 0.184 |

Answering Scheme: 1=unprepared, 2=not well prepared, 3=prepared, 4=well prepared, 5=extremely well prepared
