# Supplementary material for: Exploring the Effect of the COVID-19 Pandemic on the Dental Team: Preparedness, Psychological Impacts and Emotional Reactions
Source: Front Oral Health. 2021 Apr 29;2:669752. doi: 10.3389/froh.2021.669752 (PMC8757713; doi:10.3389/froh.2021.669752)
Supplement: Supplementary file 2 [file Table_2.DOCX]

Supplementary File: Table S2 Professional Group Membership

|  | Trainee | |  | Primary Care | |  | Total Sample | |
| --- | --- | --- | --- | --- | --- | --- | --- | --- |
|  | n | % |  | n | % |  | n | % |
| VDP | 57 | 51 |  | 0 | 0 |  | 57 | 17 |
| VDHT | 7 | 6 |  | 0 | 0 |  | 7 | 2 |
| CT1 | 10 | 9 |  | 0 | 0 |  | 10 | 3 |
| CT2 | 3 | 3 |  | 0 | 0 |  | 3 | 1 |
| CT3 | 5 | 5 |  | 0 | 0 |  | 5 | 2 |
| STr | 8 | 7 |  | 0 | 0 |  | 8 | 2 |
| GDS dentist | 0 | 0 |  | 104 | 48 |  | 104 | 32 |
| PDS dentist | 0 | 0 |  | 13 | 6 |  | 13 | 4 |
| Dental Nurse | 0 | 0 |  | 82 | 38 |  | 82 | 25 |
| Hygienist | 0 | 0 |  | 11 | 5 |  | 11 | 3 |
| Therapist | 0 | 0 |  | 1 | 0 |  | 1 | 0 |
| Hygienist Therapist | 0 | 0 |  | 6 | 3 |  | 6 | 2 |
| Orthodontic Therapist | 0 | 0 |  | 1 | 0 |  | 1 | 0 |
| Trainee DN | 19 | 17 |  | 0 | 0 |  | 19 | 6 |
| Trainee OT | 2 | 2 |  | 0 | 0 |  | 2 | 1 |
| Total | 111 | 34 |  | 218 | 66 |  | 329 | 100 |
