# Supplementary material for: Exploring the Effect of the COVID-19 Pandemic on the Dental Team: Preparedness, Psychological Impacts and Emotional Reactions
Source: Front Oral Health. 2021 Apr 29;2:669752. doi: 10.3389/froh.2021.669752 (PMC8757713; doi:10.3389/froh.2021.669752)
Supplement: Supplementary file 3 [file Table_3.DOCX]

Supplementary File: Table S3 Cronbach alpha values for psychological constructs

|  | Published values | Present Study values |
| --- | --- | --- |
| Construct |  |  |
|  |  |  |
| Impact of Event Scale (IES) |  |  |
| IES Total Score | 0.96 ‡ | 0.95 |
| IES Intrusion | 0.94 | 0.90 |
| IES Avoidance | 0.87 | 0.87 |
| IES Hyperarousal | 0.91 | 0.86 |
|  |  |  |
| Preparedness (DPPPS)‡‡ |  |  |
| P-Cope-C19 |  | 0.85 |
| P-Quality-C19 |  | 0.88 |
|  |  |  |
| Burnout |  |  |
| Emotional Exhaustions | 0.89† | 0.95 |
| Depersonalisation | 0.74 | 0.80 |
|  |  |  |
| PHQ-2 | 0.79†† | 0.83 |
|  |  |  |

† [36], †† [37], ‡[19], ‡‡ modified version of preparedness scale: no published alpha coefficients for this specific set of sub-scales [12]
