# Supplementary material for: Exploring the Effect of the COVID-19 Pandemic on the Dental Team: Preparedness, Psychological Impacts and Emotional Reactions
Source: Front Oral Health. 2021 Apr 29;2:669752. doi: 10.3389/froh.2021.669752 (PMC8757713; doi:10.3389/froh.2021.669752)
Supplement: Supplementary file 4 [file Table_4.DOCX]

Supplementary File: Table S4 Correlation matrices for Structural Equation Model (complete data for total sample and separate staff groups)

Total Sample (N=299)

|  | P-Cope-C19 | P-Qual-C19 | IES-I | IES-A | IES-H | EE | DP | PHQ1 | PHQ2 |
| --- | --- | --- | --- | --- | --- | --- | --- | --- | --- |
| P-Cope-C19 | 1 |  |  |  |  |  |  |  |  |
| P-Qual-C19 | 0.38 | 1 |  |  |  |  |  |  |  |
| IES-I | -0.48 | -0.20 | 1 |  |  |  |  |  |  |
| IES-A | -0.44 | -0.24 | 0.82 | 1 |  |  |  |  |  |
| IES-H | -0.48 | -0.24 | 0.88 | 0.77 | 1 |  |  |  |  |
| EE | -0.49 | -0.27 | 0.61 | 0.53 | 0.60 | 1 |  |  |  |
| DP | -0.20 | -0.21 | 0.46 | 0.42 | 0.43 | 0.52 | 1 |  |  |
| PHQ1 | -0.42 | -0.26 | 0.51 | 0.48 | 0.54 | 0.50 | 0.34 | 1 |  |
| PHQ2 | -0.52 | -0.21 | 0.64 | 0.57 | 0.64 | 0.58 | 0.29 | 0.71 | 1 |
|  |  |  |  |  |  |  |  |  |  |
| Trainees (N=111) | |  |  |  |  |  |  |  |  |
|  | P-Cope-C19 | P-Qual-C19 | IES-I | IES-A | IES-H | EE | DP | PHQ1 | PHQ2 |
| P-Cope-C19 | 1 |  |  |  |  |  |  |  |  |
| P-Qual-C19 | 0.40 | 1 |  |  |  |  |  |  |  |
| IES-I | -0.43 | -0.19 | 1 |  |  |  |  |  |  |
| IES-A | -0.38 | -0.20 | 0.80 | 1 |  |  |  |  |  |
| IES-H | -0.42 | -0.24 | 0.87 | 0.75 | 1 |  |  |  |  |
| EE | -0.45 | -0.39 | 0.40 | 0.30 | 0.41 | 1 |  |  |  |
| DP | -0.07 | -0.13 | 0.37 | 0.32 | 0.38 | 0.39 | 1 |  |  |
| PHQ1 | -0.26 | -0.30 | 0.39 | 0.36 | 0.42 | 0.27 | 0.24 | 1 |  |
| PHQ2 | -0.37 | -0.21 | 0.50 | 0.43 | 0.53 | 0.39 | 0.10 | 0.62 | 1 |
|  |  |  |  |  |  |  |  |  |  |
| Primary Care (N=193) | |  |  |  |  |  |  |  |  |
|  | P-Cope-C19 | P-Qual-C19 | IES-I | IES-A | IES-H | EE | DP | PHQ1 | PHQ2 |
| P-Cope-C19 | 1 |  |  |  |  |  |  |  |  |
| P-Qual-C19 | 0.35 | 1 |  |  |  |  |  |  |  |
| IES-I | -0.48 | -0.20 | 1 |  |  |  |  |  |  |
| IES-A | -0.48 | -0.26 | 0.83 | 1 |  |  |  |  |  |
| IES-H | -0.49 | -0.23 | 0.89 | 0.78 | 1 |  |  |  |  |
| EE | -0.48 | -0.20 | 0.70 | 0.65 | 0.66 | 1 |  |  |  |
| DP | -0.22 | -0.22 | 0.50 | 0.46 | 0.44 | 0.54 | 1 |  |  |
| PHQ1 | -0.50 | -0.24 | 0.57 | 0.54 | 0.59 | 0.60 | 0.38 | 1 |  |
| PHQ2 | -0.57 | -0.20 | 0.70 | 0.64 | 0.69 | 0.65 | 0.35 | 0.75 | 1 |
